# Clinical evaluation of two consanguineous families with homozygous mutations in *BEST1*

**Published:** 2011-06-16

**Authors:** Teresa Piñeiro-Gallego, María Álvarez, Inés Pereiro, Severiano Campos, Dror Sharon, Patrik Schatz, Diana Valverde

**Affiliations:** 1 Departamento de Bioquímica, Genética e Inmunología, Facultad de Biología, Universidad de Vigo, Vigo, Spain; 2 Servicio de Oftalmología, Complejo Hospitalario Universitario de Vigo, Vigo, Spain; 3 Department of Ophthalmology, Hadassah-Hebrew University Medical Center, Jerusalem, Israel; 4 Department of Ophthalmology, Lund University Hospital, University of Lund, Sweden; 5 National Eye Clinic, Kennedy Center, Glostrup, Denmark; 6 Department of Ophthalmology, Glostrup Hospital, University of Copenhagen, Denmark

## Abstract

**Purpose:**

To describe the clinical and genetic findings in two consanguineous families with Best vitelliform macular dystrophy (BVMD) and homozygous mutations in the bestrophin-1 (*BEST1*) gene.

**Methods:**

Ophthalmologic examination was performed in eight members of two families originating from Spain and Denmark. Mutation screening was performed using the Vitelliform Macular Dystrophy mutation array from Asper Biotech, and by the directed genomic sequencing of *BEST1.*

**Results:**

Two homozygous mutations were detected in these families. Mutation c.936C>A (p.Asp312Glu) has been reported previously in a Danish family; here, we describe four additional individuals in this family demonstrating findings compatible with a severe dominant BVMD, albeit with reduced penetrance in heterozygotes. In the Spanish family, a novel homozygous missense mutation in exon 4, c.388 C>A (p.Arg130Ser), was identified in the siblings. Homozygous siblings demonstrated evidence of multifocal vitelliform retinopathy, whereas heterozygous family members presented findings ranging from isolated reduction of the electrooculogram Arden ratio to normal values on all clinical parameters.

**Conclusions:**

As demonstrated in these consanguineous families, a great clinical variability is associated with homozygous mutations in *BEST1,* ranging from severe dominant BVMD with reduced penetrance in heterozygotes to autosomal recessive bestrophinopathy.

## Introduction

Bestrophin-1 is the product of the gene *BEST1*. This protein is mainly expressed in the basolateral plasma membrane of the retinal pigment epithelium (RPE) [1], although some expression has also been detected in the kidney, brain, spinal cord, and testis [2,3]. Bestrophin-1 has sequence similarity with the RFP protein family, so named for its highly conserved arginine (R), phenylalanine (F), and proline (P) amino acid sequence motif. This protein contains several domains with a high degree of evolutionary conservation [2]. Protein bestrophin-1, consisting of 585 amino acids, is required for normal ocular development [4,5]. Its function remains unclear and has been proposed to act as a Cl^−^ channel activated by intracellular Ca^2+^ and/or as a channel regulator [6].

*BEST1* has been mapped on the long arm of chromosome 11q12-q13, and was first identified in 1998 [3]. At least 253 mutations have been described in this gene (VMD2_database), and these have been related to four clinically distinguishable degenerative human eye diseases, collectively referred to as bestrophinopathies. The clinical features in patients with mutations in *BEST1* seem to cluster into at least four major categories, as reviewed by Marmorstein et al. [6]: Best vitelliform macular dystrophy or Best disease (BVMD, OMIM 153700), autosomal dominant vitreoretinochoroidopathy (OMIM 193220), autosomal recessive bestrophinopathy (ARB, OMIM 611809), and adult-onset vitelliform macular degeneration (OMIM 608161). Recently, missense mutations in *BEST1* have been implicated in retinitis pigmentosa (RP) [7].

Burgess et al. [8] were the first to describe a distinct retinal disorder they designated as autosomal recessive bestrophinopathy (ARB). Characteristics of the disorder included central visual loss with progressive decrease in visual acuity, a characteristic retinopathy, an absent electrooculogram (EOG) light rise, and a reduced electroretinogram (ERG). None of the patients showed the vitelliform lesions typical of Best disease, but showed a diffuse irregularity of the reflex from the RPE, including dispersed punctate flecks. All patients showed an accumulation of fluid within and/or beneath the neurosensory retina in the macula. All patients were hyperopic, and three patients from two families also had angle-closure glaucoma [8].

To date, only 13 patients from 11 different families have been described [7-14] with biallelic mutations in *BEST1*. Most of these mutations have been described as missense mutations, except for a homozygous R200X nonsense or stop mutation (in two patients from the same family) and a heterozygous L88del17 deletion (in one patient). In addition, two individuals from a single family were described as having atypical BVMD [15], although the description of the phenotype is suggestive of ARB. Furthermore, a large survey by Kinnick et al. has recently identified 11 other patients with autosomal recessive macular dystrophy and a range of different biallelic mutations in *BEST1*, including intronic mutations and deletions [16].

In the present study, we describe two consanguineous families with homozygous mutations in *BEST1*, being the sixth and seventh patients characterized until now with homozygous mutations in this gene.

## Methods

### Patients

**Figure 1:**
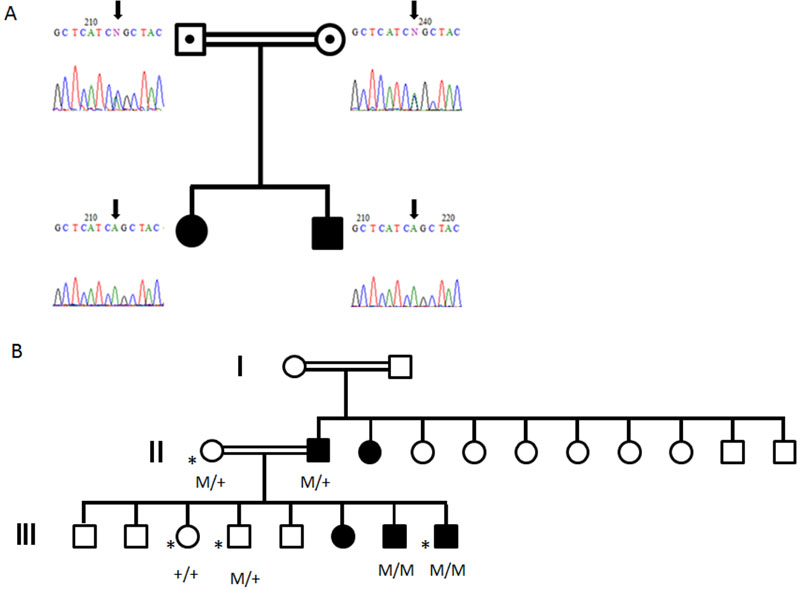
Cosegregation analysis of the mutations. **A**: Pedigree of the Spanish family and segregation analysis of the p.Arg130Ser mutation of *BEST1*. The siblings were homozygous for p.Arg130Ser while the parents demonstrate heterozygosity for the mutation. Double lines indicate first degree cousins and symbols with a dot inside indicate carry individuals for the mutation. **B**: Pedigree of the Danish family with *BEST1* mutations, modified from Schatz et al. [12]. In the present study, four additional family members were examined, marked by an asterisk. M/+ denotes heterozygosity for mutations in *BEST1,* whereas M/M denotes homozygosity.

We report two consanguineous (first-degree cousin) families. A Spanish family (S) with two siblings affected (a 4-year-old girl and a 9-year-old boy) and a Danish family with two siblings affected (a 6-year-old and a 10-year-old boy). The individual clinical details are summarized in Table 1, and pedigrees are shown in Figure 1. The recruitment of the Spanish patients and relatives was performed through the Servicio de Oftalmología of the Complejo Hospitalario Universitario de Vigo (Pontevedra, Spain).

**Table 1:** Clinical description of the homozygous patients from Spanish family and Danish family.

**Family**	**Individual**	**Age of onset**	**VA (Snellen)/ refraction**	**Retina**	**Fluorescein angiography**	**ERG**	**EOG Arden ratio**
S	Female sibling	4 years	0.8–0.8 (Pigassou)	OD: pseudohypopion macular lesion. OS: retractile-atrophic lesion. Multifocal subretinal deposits of vitelliform material and diffuse alterations of RPE in posterior pole and peripheral retina in both eyes.	OD: macular hypo-fluorescent lesions due to deposits of vitelliform material and hyper-fluorescent lesions due to atrophic lesions of the RPE. OS: hyper-fluorescent lesion due to its retractile-atrophic stage. Hyper-fluorescent lesions in the periphery of both eyes.	Normal	Reduced OD: 1.00 OS: 1.12
S	Male sibling	9 years	0.6–0.6 (Snellen)	Perimacular scars. Multifocal vitelliform discs in peripheral retina.	Hyper-fluorescent perimacular lesions. Multifocal peripheral hypofluorescent disc-like lesions.	Reduced (a and b waves amplitudes)	Reduced OD:1.03 OS:1.12
DII	III8	6 years	0.7–0.7 (Snellen)	Slight vitelliform macular degeneration	Not examined	Not examined	Not possible
DII	III7	9 years	0.5–0.4 (Snellen)	Multifocal vitelliform retinopathy.	Described in Schatz et al. [12]	Reduced	Not possible

VA, visual acuity; OD, right eye; OS, left eye.

Clinical and molecular genetic findings in two other individuals from the Danish family (DII) have been described previously [14]. This molecular study adhered to the tenets of the Helsinki Declaration. Informed consent was obtained from all patients and relatives after explanation of the nature and possible consequences of the study. Approval for the study was obtained from an ethics committee. The Spanish control group consisted of 50 unrelated normal individuals provided by the Complejo Hospitalario Universitario de Vigo.

### Clinical evaluation

#### Spanish family

Clinical evaluation consisted of Pigassou and Snellen best-corrected visual acuity test, slit lamp biomicroscopy, extrinsic ocular motility, retinography, fluorescein angiography (AGF), fundus autofluorescence (FAF), optical coherence tomography (OCT), visual evoked potentials (VEP), EOG, and full-field ERG. The Pigassou test was used to evaluate the female sibling’s visual acuity (under five years old) and Snellen test was used to value the male sibling’s visual acuity (over five years old). Fundus photography and AGF were recorded using the Topcon TRC SOIA Retinal Camera together with a HAD Color Video Camera connected to a Topcon Digital Imagenet 2000 System (Newbury Berkshire, UK). Autofluorescence was recorded using the Carl Zeiss Meditec AG FF 450 plus a Dolphin IR Digital Interface (Carl Zeiss Meditec, Jena, Germany). OCT was performed using a Cirrus TM HD-OCT Spectral Domain together with a scanning laser fundus camera (Carl Zeiss®). ERGs, EOGs, and visual evoked responses were recorded using a Medelec Synergy (Oxford® Instruments, Oxon, England).

#### Danish family

Multifocal ERGs were recorded using a visual evoked response imaging system (VERIS 4; EDI, San Mateo, CA), as described previously [17].

In family DII, fundus morphology was examined using a combined OCT and scanning laser fundus camera (Spectralis HRA-OCT, Heidelberg Engineering, Heidelberg, Germany). EOG was recorded with a Nicolet analysis system (Nicolet Biomedical Instruments, Madison, WI), as described previously [18,19].

### Molecular methods

Molecular genetic findings in two of the individuals from the Danish family have been described previously [14]. In this study, four additional family members were examined (Figure 1). Blood was collected by venipuncture from members of the Spanish family, and genomic DNA was isolated with Flexi Gene DNA Kit (Qiagen, Hilden, Germany) following the instructions of the manufacturer for isolation of DNA from 4 to 14 ml whole blood.

The DNA samples were analyzed using the Vitelliform Macular Dystrophy (BEST-VMD) mutation array from Asper Biotech (Asper Ophthalmics, Tartu, Estonia). The BEST-VMD test has been developed for screening 138 mutations/SNPs from the *BEST1* (VMD2) gene. This array utilizes Arrayed Primer Extension technology, first described in 1996 [20].

The 11 exons of the *BEST1* gene were amplified by PCR in a MJ MiniTM Gradient Thermal Cycler (Bio-Rad, Hercules, CA). The primers used (Table 2) have been previously described [2]. The sequencing reactions were precipitated, dried and analyzed on an AB 3130 genetic analyzer (Applied Biosystems, Foster City, CA). Sequence data was aligned to reference GenBank cDNA sequence NG_009033 and examined for sequence variations. Detected mutations were confirmed by a second independent PCR reaction and were identified in both forward and reverse strands.

**Table 2:** Oligonucleotides for genomic amplification and sequencing of *BEST1* gene.

**Exon**	**Forward primer (5′-3′)**	**Reverse primer (5′-3′)**	**Tm (°C)**	**Product size (pb)**
1	AGCCCAACACCCTCCAAGAA	AAGGTGCCCCAGGTGGACT	58	297
2	CCCTACAAACCCCCAATCG	CTGGGAAGGATGTGGCTGG	62	272
3	AGTCTCAGCCATCTCCTCGC	CAGGCTCCAGACAGGCCA	63	212
4	AGAAAGCTGGAGGAGCCGA	AGGAATGGAAGATGGGTGGA	61	413
5	GCCATCCCTTCTGCAGGTT	GTACAGACAGGGCTGCCGC	58	239
6	GGGGCAGGTGGTGTTCAGA	CTAGGCTGGTGAGGCTGCC	62	150
7	TGATTTCAGGGTTCCCACCTAG	AGCTGCCTGAGACGAGGATG	58	286
8	AGGGTTTACAGAGCCTCACCTG	CATGGACCTTCCCCAAAGTG	62	158
9	CCTCCAAGTCATCAGGCACATA	CTAGTGCAGGGGTCTGCCTAG	58	289
10A	ACTGGCTCAGCCCTGCATC	CAGGCTACTACAGTGCCCCAC	62	340
10B	CCTTCAAGTCTGCCCCACTG	GAACCAGGGGCACTGCA	62	417
11	GGGACCTTCCATACTTATGCTG	TTCATCCCAAGACCT	61	492

We used two sequence homology–based tools—Protein Basic Local Alignment Search Tool (BLASTP) and Polymorphism Phenotype (PolyPhen)—to predict the potential impact of two amino acid substitutions.

## Results

### Spanish family

**Figure 2:**
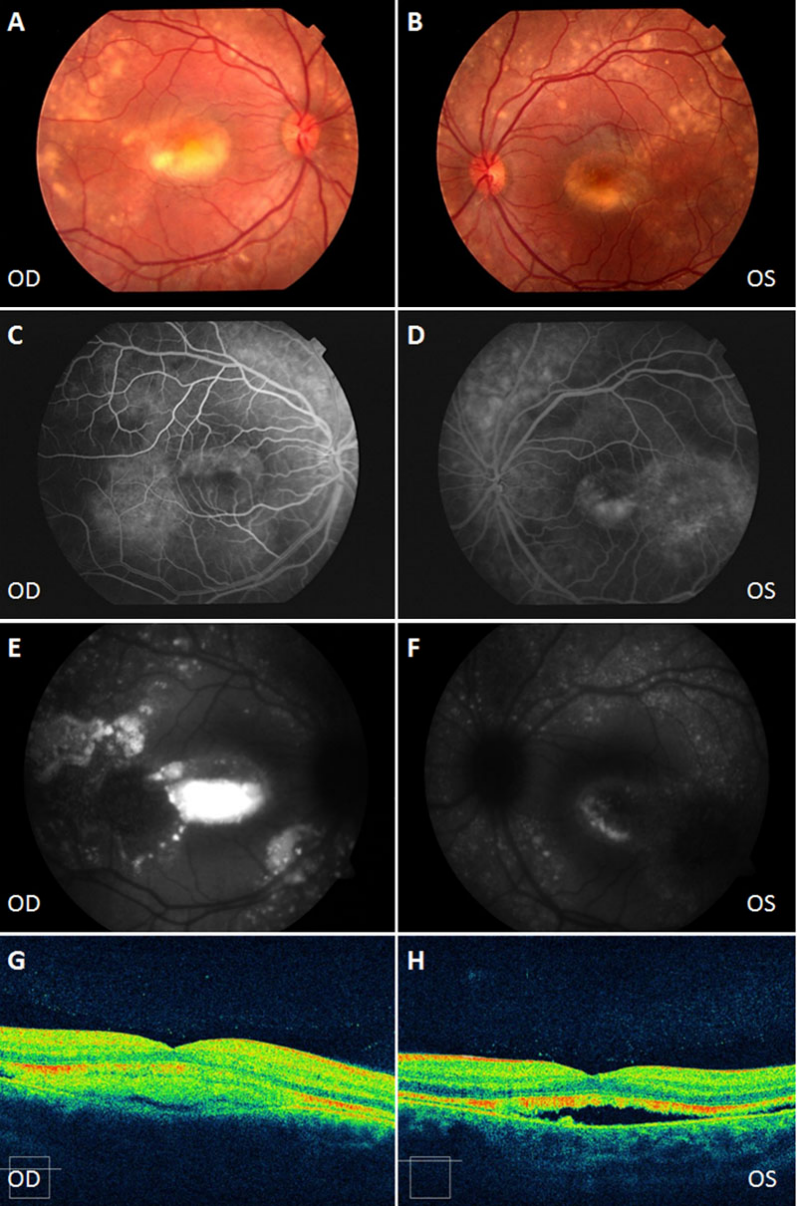
Clinical evaluation of the female sibling from the Spanish family. Color fundus appearance photographs (**A**, **B**). The right eye shows a typical pseudohypopyon stage with a deposit of yellowish vitelliform material in the lower half of the macular lesion. The left eye shows a characteristic atrophic stage. Fluorescein angiography (AGF) shows a hyperfluorescence window defect due to atrophy of the retinal pigment epithelium (RPE; **C**, **D**), and hypofluorescence due to a deposit of lipofuscin (**C**). Vitelliform lesions appear as areas of hyperautofluorescence (**E**) and hypoautofluorescence (**F**) fundus autofluorescence (FAF). Optical coherence tomography (OCT) section of the pseudohypopyon lesion shows a hyperreflective material beneath the retina in the right eye (**G**) and a neurosensory retinal detachment in the left eye (**H**). OD: Right eye, OS: Left eye.

Two siblings, a 4-year-old female and a 9-year-old male, and their parents were studied. Both siblings had vitelliform macular dystrophy. The female presented a pseudohypopyon in the right eye and predominantly atrophic changes in the left eye (Figure 2). Visual acuity was 0.8 in both eyes; the patient was moderately hyperopic and had accommodative esotropia. OCT demonstrated a neurosensory retinal detachment, typical of this stage (Figure 2). Autofluorescence imaging showed hyperautofluorescent lesions that represented the deposit of vitelliform material in the posterior pole and peripheral retina, and hypoautofluorescent lesions that represented RPE atrophy (Figure 2). The EOG Arden ratio was reduced (OD:1.00/OS:1.12), while full-field ERG and VEP were normal.

**Figure 3:**
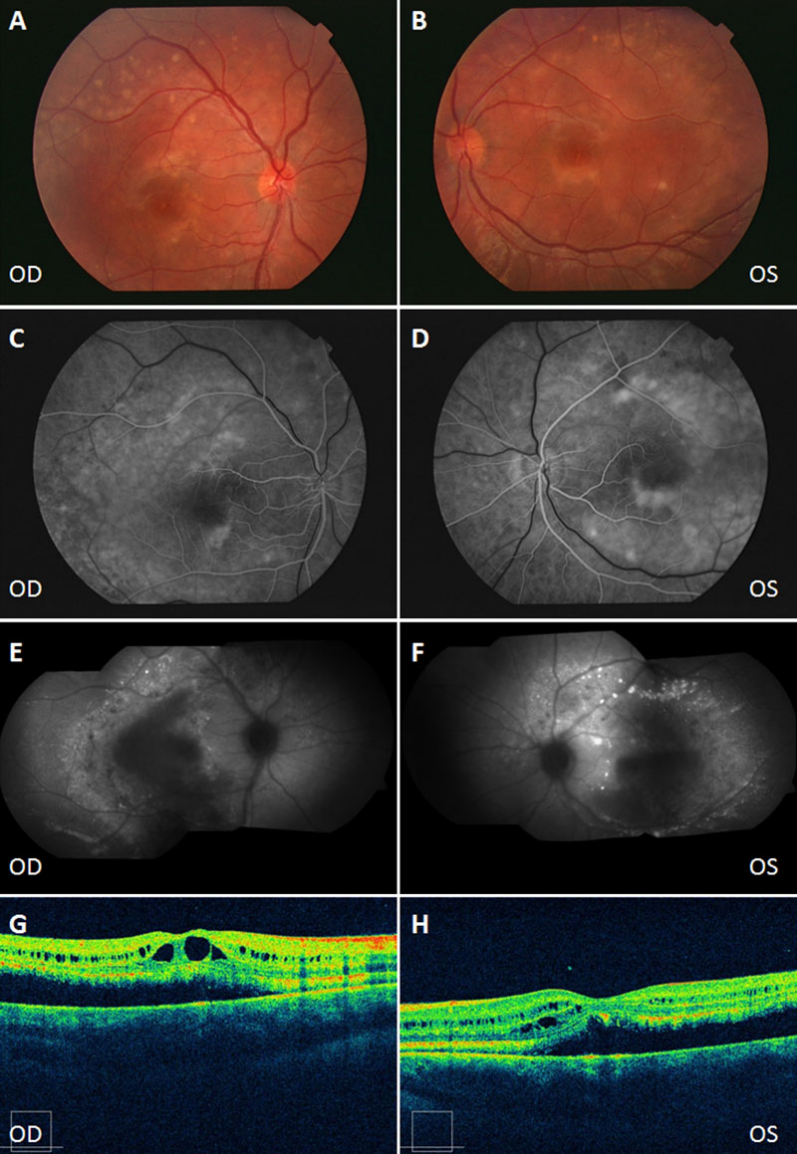
Clinical evaluation of the male sibling from the Spanish family. Color fundus appearance photographs showing perimacular scars in both eyes (**A**, **B**). By fluorescein angiography (AGF), there are multiple hypofluorescent discs in the peripheral retina caused by the deposition of vitelliform material. AGF also shows a hyperfluorescence window defect due to atrophy of the retinal pigment epithelium (RPE). Multiple hypoautofluorescent lesions in the peripheral retina due to lipofuscin deposits on the fundus autofluoresence image (**E**, **F**). Optical coherence tomography (OCT) showing RPE detachment and cystic degeneration of the sensory retina, typical of advanced stages of retinal dystrophies (**G**, **H**). OD: Right eye, OS: Left eye.

The male presented perimacular scars in both eyes. By AGF, there were multiple hypofluorescent discs in the peripheral retina caused by the deposition of vitelliform material. The OCT showed cystic degeneration of the sensory retina, typical of advanced stages of retinal dystrophies (Figure 3). In this case, not only the EOG Arden ratio (OD:1.03/OS:1.12), but also the ERG was altered (reduced amplitudes of the *a* and *b* waves). Nevertheless, the patient had good visual acuity (0.6 in both eyes). The VEP was normal. The mother presented with a slightly reduced EOG Arden ratio in the right eye (OD:1.73/OS:2.00), but normal fundoscopy, and clinical examination of the father was normal on all investigations (retinography, FAF, OCT, VEP, EOG, and ERG).

Screening for previously reported *BEST1* mutations using the BEST-VMD array was negative. Nevertheless, this array has a great potential for a first screening diagnosis as some of the disease-causing mutations are recurrent. Sequencing of *BEST1* in the affected siblings revealed a novel homozygous missense mutation in exon 4, c.388C>A. This mutation replaces arginine with serine at codon 130 (p.Arg130Ser). Additional non-disease-causing polymorphisms were also identified. The proband’s parents were heterozygous for the c.388C>A mutation. The mutation was absent in 50 healthy unaffected European controls.

Simulation for functional changes by a structure homology-based method using the Polyphen program resulted in classifying the Arg130Ser change as probably damaging (Position-Specific Independent Counts, PSIC02.615). Arg130Ser is with high confidence supposed to affect protein function or structure.

**Figure 4:**
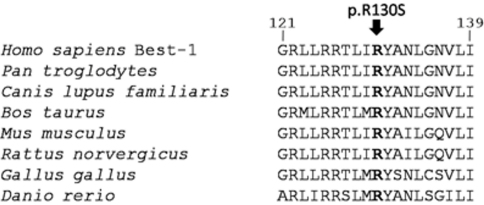
Multiple sequenced alignment (Basic Local Alignment Search Tool) of bestrophin-1 around the mutated residue (p.Arg130Ser) for eight species. The residues at position 130 are highly conserved.

Multiple alignment of bestrophin-1 protein (BLASTP) showed conservation of the regions that harbor the missense variant p.Arg130Ser (Figure 4). The residue in position 130 is located in the N-terminal half of the protein, which shows the highest evolutionary conservation [21,22].

### Danish family

The c.936C>A transversion, leading to p.Asp312Glu, is a novel mutation found in family DII, in which the affected father was heterozygous while his affected son III:7 was homozygous for this dominant mutation [14]. Amino acid 312 is located in a highly conserved region of bestrophin, is perfectly preserved in different species, and mutations in many amino acids flanking it cause Best disease, adult-onset vitelliform macular degeneration, or ARB. Furthermore, a pathologically reduced EOG Arden ratio in the presence of a normal full-field ERG in the heterozygous father of family DII indicated that p.Asp312Glu is a disease-causing mutation leading to AD Best disease [14].

**Figure 5:**
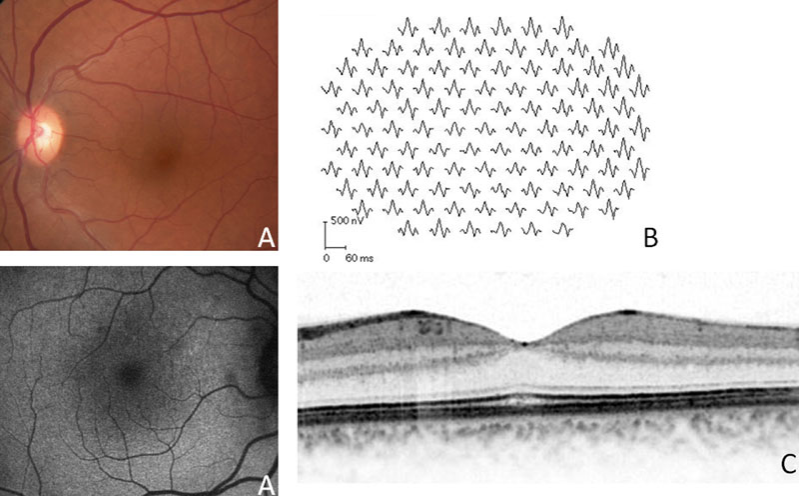
Clinical evaluation of patient II:1 from the Danish family. Patient II:1 showed normal fundus appearance (**A**), multifocal-electroretinograms (ERGs; **B**), and optical coherence tomography (OCT; **C**).

**Figure 6:**
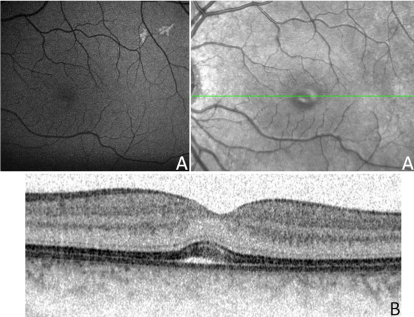
Clinical evaluation of patient III:8 from the Danish family. Fundus appearance (**A**) and optical coherence tomography (OCT) showing a tiny vitelliform foveal lesion (**B**).

The mutation was subsequently verified heterozygously in the clinically unaffected mother II:1 (Figure 5) and son III:4, both of whom showed normal fundi, OCT, and autofluorescence, but whose EOG Arden ratios were pathologically reduced (1.29/1.39 and 1.57/1.39, respectively). The mutation was also found homozygously in two clinically affected sons III:7 and III:8, of which the former has been described recently by Schatz et al. [14]. The latter, III:8, showed a tiny vitelliform foveal lesion, which was visualized by OCT (Figure 6). EOG was not possible due to the young age (six years old). The mutation p.Asp312Glu was absent in the clinically unaffected daughter III:3.

## Discussion

Over 253 different *BEST1* mutations have been described. There is great phenotypic variability among patients carrying these mutations, including those clinically diagnosed with BVMD, AVMD, autosomal dominant vitreoretinochoroidopathy, RP, and ARB. Burgess clinically described this last condition in five families with biallelic mutations in the *BEST1* gene [8].

In the current study, the clinical examination showed a different phenotype in the two siblings from the Spanish family and in at least one sibling from the Danish family. In the Spanish family, the female sibling showed macular lesions, typical of Best disease, and hyperautofluorescent lesions in the posterior pole and midperiphery. The male sibling could be classified as exhibiting a late stage of Best with perimacular scars, also featuring multifocal disease. In the Danish family, the two homozygous boys demonstrated evidence of multifocal vitelliform lesions, at least by FAF, although the findings were subtle in the youngest boy III:8 and severe in III:3. In neither of them was it possible to obtain an EOG.

Patients with biallelic mutations in *BEST1* show a highly variable phenotype [7-16]. In many cases, the phenotypes described are associated with ARB; however, in other cases these are more to similar to BVMD. To date, only a few homozygous cases have been identified. Bakall et al. [9] described a patient homozygous for the p.W93C mutation whose disease was as severe as that reported for affected family members heterozygous for the same mutation. This differs from the study of Querques et al. [11], who reported an individual homozygous for the p.R92C mutation with a phenotype consistent with the diagnosis of multifocal BVMD, considered more severe than that reported for heterozygotes. Nevertheless, in the Spanish family, the parents heterozygous for p.Arg130Ser were clinically unaffected and only the mother showed a slightly reduced EOG Arden ratio in the right eye (OD: 1.73), which was considered nonpathological [23]. In this case, unlike Querques et al.’s report, the correct segregation of the mutation excludes a deletion or hemizygosity. In the Danish family, the heterozygous father demonstrated a reduced EOG Arden ratio of 1.4 and an atypical macular degeneration characterized mainly by atrophy [14]. The mother was clinically unaffected and presented an EOG Arden ratio of 1.29 and 1.39 in the right and left eye, respectively. The pathology described in the father could be the result of overlapping ocular entities; however, a screening for mutations in *ABCR* was negative [14].

In all these cases presented, the severity of the phenotype seems to be associated to the homozygous state of the mutation in *BEST1.*

Burgess et al. [8] identified another two individuals homozygous (R200X) for *BEST1* described as a typical ARB phenotype, and finally Davidson et al. presented an affected RP individual homozygous (L140V) for *BEST1*.

The review of the literature showed two cases that could be suspected to be biallelic for *BEST1.* Boon et al. [24] described a five-year-old patient with a mutation (p.Lys194_Ala195insVal) in *BEST1*. The father was heterozygous for the same mutation, displayed no abnormalities in eye examination, and had a normal EOG. This case could involve a recessive inheritance where the second mutation was not detected. Caldwell et al. [25] described an eight-year-old patient heterozygous for the p.E300D mutation, whose mother was also heterozygous for this mutation but was clinically unaffected. It would be very interesting to reinvestigate these cases and to establish the segregation in these families to define the dominant or recessive pattern of inheritance.

Many of the disease-causing mutations in *BEST1* cause Cl^-^ channel dysfunction. The mutations located in the highly acidic region of the C-terminus render bestrophin-1 nonfunctional as a Cl**^-^** channel, and these mutations may exert dominant negative effects. The mutations disrupt the interaction between the N and the C termini, and may disrupt the bestrophin multimer. For mutations located between the TM2 and TM5 region, including p.Arg130Ser, located in TMD3, it is not clear how the mutations affect channel function [26]. But whether or not the light peak is generated by the putative bestrophin-1 Cl^−^ channel is controversial, as some patients (e.g., the Spanish family) with certain disease-causing mutations have normal or nearly normal EOGs.

The pathological nature of the p.Arg130Ser mutation was established by virtue of 1) its absence in 50 healthy unaffected controls, 2) its cosegregation with disease in the family, 3) its Polyphen PSIC score (2.615), which suggests that it may have a functional impact, and 4) its affect on a conserved residue of the TMD3 domain of bestrophin-1 in the two proposed models for the topological organization of this protein [21,22].

This change is located outside of the different clusters of hot-spots defined for BVMD (6–30, 80–104, 221–243, and 293–312 amino acid regions) [27]. For mutations p.R141H, p.A146K, and p.P152A located in the surroundings of p.Arg130Ser, a reduced measurable basal Cl^-^ current has been demonstrated, indicating no dominant negative effect. As the heterozygous parents are unaffected, the missense change p.Arg130Ser would not be expected to have a dominant effect. In the Danish family with the c.936C>A transversion leading to p.Asp312Glu, there were three heterozygous individuals. The heterozygous father had an atypical central foveal bilateral atrophy [14] with reduced EOG Arden ratio, whereas the heterozygous mother and heterozygous brother had a normal fundus, OCT, and FAF, but the EOG Arden ratio was in the pathological range. It has been proposed by Hartzell et al. [23] that the association of bestrophin-1 with accessory subunits could alter their biophysical properties. Mutations in accessory subunits, or modifier genes, could influence the activity of bestrophin-1. It is very important to perform functional studies that shed light on the activity of this mutated protein.

The p.Arg130Ser and p.Asp312Glu changes in the homozygous state could confer a phenotype similar to BVMD, more severe than that described by Burgess et al. [8] for ARB. The varying expression and incomplete penetrance of this disease have been described by several studies. Little is known about the exact mechanism of the reduced penetrance and variable expression, so several explanations remain possible.

As pointed out by Marmorstein and coworkers [6], if ARB is a true null phenotype, it would be predicted to resemble a severe case of BVMD, as has been found in homozygous individuals in the Spanish family. On the other hand, in the Danish family, the presented information regarding the additional investigated family members is compatible with a severe dominant BVMD, albeit with reduced penetrance in heterozygotes.

In summary, we present evidence of great clinical variability associated with homozygous mutations in *BEST1*. Other factors, genetic or environmental, could modulate the phenotype in these families, since the retinopathy in the presented patients is not identical even within the same families.
